# Controlling Immune Rejection Is a Fail-Safe System against Potential Tumorigenicity after Human iPSC-Derived Neural Stem Cell Transplantation

**DOI:** 10.1371/journal.pone.0116413

**Published:** 2015-02-23

**Authors:** Go Itakura, Yoshiomi Kobayashi, Soraya Nishimura, Hiroki Iwai, Morito Takano, Akio Iwanami, Yoshiaki Toyama, Hideyuki Okano, Masaya Nakamura

**Affiliations:** 1 Department of Orthopaedic Surgery, Keio University School of Medicine, 35 Shinanomachi, Shinjuku, Tokyo, 160-8582, Japan; 2 Department of Physiology, Keio University School of Medicine, 35 Shinanomachi, Shinjuku, Tokyo, 160-8582, Japan; University of California, San Diego, UNITED STATES

## Abstract

Our previous work reported functional recovery after transplantation of mouse and human induced pluripotent stem cell-derived neural stem/progenitor cells (hiPSC-NS/PCs) into rodent models of spinal cord injury (SCI). Although hiPSC-NS/PCs proved useful for the treatment of SCI, the tumorigenicity of the transplanted cells must be resolved before they can be used in clinical applications. The current study sought to determine the feasibility of ablation of the tumors formed after hiPSC-NS/PC transplantation through immunoregulation. Tumorigenic hiPSC-NS/PCs were transplanted into the intact spinal cords of immunocompetent BALB/cA mice with or without immunosuppressant treatment. *In vivo* bioluminescence imaging was used to evaluate the chronological survival and growth of the transplanted cells. The graft survival rate was 0% in the group without immunosuppressants versus 100% in the group with immunosuppressants. Most of the mice that received immunosuppressants exhibited hind-limb paralysis owing to tumor growth at 3 months after iPSC-NS/PC transplantation. Histological analysis showed that the tumors shared certain characteristics with low-grade gliomas rather than with teratomas. After confirming the progression of the tumors in immunosuppressed mice, the immunosuppressant agents were discontinued, resulting in the complete rejection of iPSC-NS/PC-derived masses within 42 days after drug cessation. In accordance with the tumor rejection, hind-limb motor function was recovered in all of the mice. Moreover, infiltration of microglia and lymphocytes was observed during the course of tumor rejection, along with apoptosis of iPSC-NS/PC-generated cells. Thus, immune rejection can be used as a fail-safe system against potential tumorigenicity after transplantation of iPSC-NS/PCs to treat SCI.

## Introduction

Enormous progress has been made in the field of regenerative medicine centered on cell transplantation therapy, largely owing to advances in stem cell biology. For example, we recently reported the efficacy of human induced pluripotent stem cell (hiPSC)-derived neural stem/progenitor cell (hiPSC-NS/PC) transplantation for the treatment of spinal cord injury (SCI) in rodents and a non-human primate, the common marmoset [1–4]. However, concerns about the potential tumorigenicity of iPSCs and their progeny must be addressed before these cells can be used in clinical practice.

To pursue the issue of iPSC-NS/PCs safety, the cells must be thoroughly characterized. To do this, the expression of cell surface markers and differentiation-associated genes, genome copy number variation, and DNA methylation status should be analyzed using flow cytometry, microarray technology, and related techniques [4]. Furthermore, the tumorigenicity of iPSC-NS/PCs requires careful evaluation by grafting the cells into immunodeficient mouse models. However, even these quality control measures prior to clinical cell transplantation cannot completely exclude the possibility of late-onset tumorigenesis. Thus, a fail-safe strategy against tumorigenesis is essential. Studies using the Herpes Simplex Virus type 1 thymidine kinase (HSV/TK) system for the selective ablation of stem cell-derived tumors reported a reduced cancer risk after the transplantation of mouse embryonic stem cells (ESCs) and iPSCs into animal models [5,6]. Furthermore, an inducible caspase 9 system is already in clinical use, although it has not been applied to stem cells [7]. However, because the HSV/TK system is accompanied by issues of genomic insertion, the establishment of an anti-tumor system with higher safety remains of utmost importance.

Previous reports suggested that the optimal timing of cell transplantation for SCI is at the subacute phase, when the inflammatory response has subsided, but before the formation of the glial scar is complete (generally 2–4 weeks after SCI in non-human primates and rodents) [8,9]. Given the limitations of this therapeutic time window, autologous transplantation of iPSC-NS/PCs for SCI is technically challenging at present [4,10,11]. Furthermore, vigorous validation and quality control of each iPSC lines and its derivatives are necessary for their clinical use. This would involve the expansion, derivation, and quality control of patient-specific iPS-NSCs, and is therefore too time-consuming and expensive to treat acute and sub-acute SCI patients. Therefore, clinical application of iPS-NSCs for SCI will presumably necessitate allogeneic procedures in the foreseeable future.

Compared with other organ systems, the central nervous system (CNS), including the spinal cord, is regarded as a relatively “immune-privileged” site, signifying that the CNS is immunologically tolerant [12–16]. Moreover, the ability of NS/PCs to modulate the immune response by secreting immunosuppressive cytokines (e.g., transforming growth factor-β1) has been described both *in vitro* and *in vivo* [17–19]. However, as evidenced by the grafting of rat NS/PCs into the lesioned rat spinal cord, the T-cell-mediated immune response can still be induced in the host following the transplantation of allogeneic cells [20]. Therefore, to prevent the chronic rejection of grafted cells and to promote their long-term engraftment, combinatorial immunosuppressive/cell transplantation therapy is required for a certain period of time following SCI.

The present study explored the xenotransplantation of tumorigenic hiPSC-NS/PCs into a mouse spinal cord with or without immunosuppressant agents. Furthermore, upon transplantation of the tumorigenic hiPSC-NS/PCs, we investigated whether the resultant stem cell-derived tumors could be eliminated by immune rejection following the withdrawal of the immunosuppressants.

## Materials and Methods

### Cell culture, neural induction, and lentivirus transduction

Cell culture and neural induction of hiPSCs (hiPSC clone 253G1[21], Caucasian, 36 years old, female, human dermal fibroblast) were performed as previously described [1,2,22,23], with slight modifications. hiPSCs (253G1) were grown on gelatin-coated (0.1%) culture dishes and irradiated murine embryonic fibroblasts (MEFs), maintained in standard ES cell medium, and used for EB formation as described previously. Thirty days after their formation, EBs were enzymatically dissociated into single cells and cultured in suspension in serum-free media hormone mix (MHM) medium for 12 days to allow neurospheres to form. The recombinant human immunodeficiency virus type 1 (HIV-1)-based lentivirus was prepared and transduced into hiPSC-derived neurospheres according to previously published methods [23]. Briefly, primary hiPSC-derived neurospheres were first dissociated and infected with a fusion HIV-1-based lentiviral vector (kindly gifted by Dr. Hara, Brain Science Institute, RIKEN, Japan) expressing ffLuc (Venus fused to firefly luciferase) [24] under the control of the elongation factor (EF) promoter (pCS II-EF-dVenus-Luc2). This vector enabled the grafted cells to be detected as strong bioluminescent ffLuc signals in live mice and as fluorescent Venus signals by using an anti-green fluorescent protein (GFP) antibody in fixed spinal cord sections because the Venus protein was originally modified from GFP. The primary hiPSC-derived neurospheres were passaged into secondary and tertiary neurospheres, including hiPSC-NS/PCs, and used for transplantation into the murine spinal cord, as described below.

### Cell transplantation

Female 8-week-old immunocompetent BALB/cA mice (20–22 g, n = 27) were anesthetized with an intraperitoneal (i.p.) injection of ketamine (100 mg/kg) and xylazine (10 mg/kg). After laminectomy at the 10^th^ thoracic spinal vertebra, the dorsal surface of the dura mater was exposed. hiPSC-NS/PCs (5 × 10^5^ cells/2 μl) were transplanted into the spinal cord with a glass micropipette at a rate of 1 μl/min using a 25 μl Hamilton syringe and a stereotaxic microinjector (KDS 310, Muromachi Kikai Co., Ltd., Tokyo, Japan). For immunosuppression, the female BALB/cA mice were randomized to receive FK506 (Prograf; Astellas Pharma US, Inc., Northbrook, IL, USA) plus anti-mouse CD4 monoclonal antibody (anti-mCD4 mAb; BioXcell, West Lebanon, NH, USA). FK506 was administered by subcutaneous injection at a dose of 5 mg/kg once daily beginning on the first day of cell transplantation, and anti-CD4 mAb was administered by i.p. injection at a dose of 10 mg/kg beginning 2 days before cell transplantation and continuing once per week after transplantation (with immunosuppressant (IS) group n = 5, IS off group n = 16).

All experiments were performed in accordance with the Guidelines for the Care and Use of Laboratory Animals of Keio University (Assurance No. 13020) and the Guide for the Care and Use of Laboratory Animals (National Institutes of Health, Bethesda, MD, USA). All surgery was performed under anesthesia, and all efforts were made to minimize animal suffering and were used humane endpoints.

### Bioluminescence imaging

The Xenogen-IVIS spectrum cooled charge-coupled device optical macroscopic imaging system (Caliper Life-Sciences, Hopkinton, MA, USA) was used for bioluminescence imaging (BLI) to confirm the survival of the transplanted hiPSC-NS/PCs. Monitoring was performed at a frequency of 1–2 times per week after cell transplantation. Briefly, D-luciferin (Promega, Madison, WI, USA) was administered via i.p. injection at a dose of 300 mg/kg body weight. Animals were placed in a light-tight chamber, and photons emitted from the luciferase-expressing cells were collected with integration times of 5 s to 2 min, depending on the intensity of the bioluminescence emission. BLI signals were quantified in maximum radiance units (photons per second per centimeter squared per steradian (p/s/cm^2^/sr)) and presented as log10 (photons per second).

### Histological analysis

Animals were anesthetized and transcardially perfused with 0.1 M phosphate buffered saline containing 4% paraformaldehyde. The spinal cords were removed, embedded in Optimal Cutting Temperature compound (Sakura Finetechnical Co., Ltd., Tokyo, Japan), and sectioned in the sagittal plane on a cryostat (Leica CM3050 S, Leica Microsystems, Buffalo Grove, IL, USA). Sections were stained with hematoxylin-eosin (HE), Hoechst 33258 dye (10 μg/mL; Sigma Chemical Co., St. Louis, MO, USA), and the following primary antibodies: anti-GFP (rabbit IgG, 1:200; Frontier Institute Co., Ltd., Hokkaido, Japan), anti-β-tubulin isotype III (mouse IgG, 1:1,000; Sigma Chemical Co.), anti-glial fibrillary acidic protein (anti-GFAP, rabbit IgG, 1:200; Dako, Carpinteria, CA, USA), anti-Oligo-1 (mouse IgG, 1:200; R&D Systems, Minneapolis, MN, USA), anti-human-specific nestin protein (rabbit IgG, 1:200; described previously [25,26]), anti-Ki-67 (rabbit IgG, 1:200; Novocastra, Newcastle upon Tyne, UK), anti-CD11b (rat IgG, 1:200; BD Pharmingen, San Diego, CA, USA), anti-terminal deoxynucleotidyl transferase (anti-TdT; included in the Apop Tag Plus Fluorescein In Situ Apoptosis Detection Kit; Chemicon, Temecula, CA, USA), and anti-CD3 (rat IgG, 1:100; AbD Serotec, Raleigh, NC, USA). Samples were examined on an inverted fluorescence microscope (BZ 9000; Keyence Co., Osaka, Japan) or a confocal laser scanning microscope (LSM 700, Carl Zeiss, Jena, Germany). To quantify the human nuclear antigen (HNA)-, Ki-67-, CD11b-, CD3-, NKp46-, and TdT-positive cells, three representative mid-sagittal sections were selected and five regions within 1 mm rostral and caudal to the lesion epicenter were automatically captured at 200× magnification. The numbers of marker-positive cells were counted in each section (n = 2 per group).

### Flow cytometric analysis

Isolated peripheral blood leukocytes were analyzed by triple immunofluorescence staining, followed by flow cytometry. The following primary antibodies were purchased from eBiosciences (San Diego, CA, USA): anti-allophycocyanin (APC)-labeled CD3 (clone 145-2C11), anti-fluorescein isothiocyanate (FITC)-labeled CD4 (clone GK 1.5), anti-phycoerythrin (PE)-labeled CD8 (clone 53–6.7), Armenian Hamster IgG Isotype Control APC (clone eBio299Arm), Rat IgG2b K Isotype Control FITC (eB149/10H5), and Rat IgG2a K Isotype Control PE (eBR2a). The cells were stained with a mixture of the primary antibodies at 4°C for 30 min. Flow cytometry was performed on a fluorescence- activated cell sorting (FACS) Calibur instrument (BD Biosciences, San Jose, CA, USA).

### Statistical analysis

All data are presented as the mean value ± the standard error of the mean. Friedman’s test followed by Dunn’s post-hoc test was used to determine significant differences in the BLI analysis. For all statistical analyses, the significance level was set at *p* < 0.05.

## Results

### Characterization of lentivirally transduced hiPSC-NS/PCs

**Fig 1:**
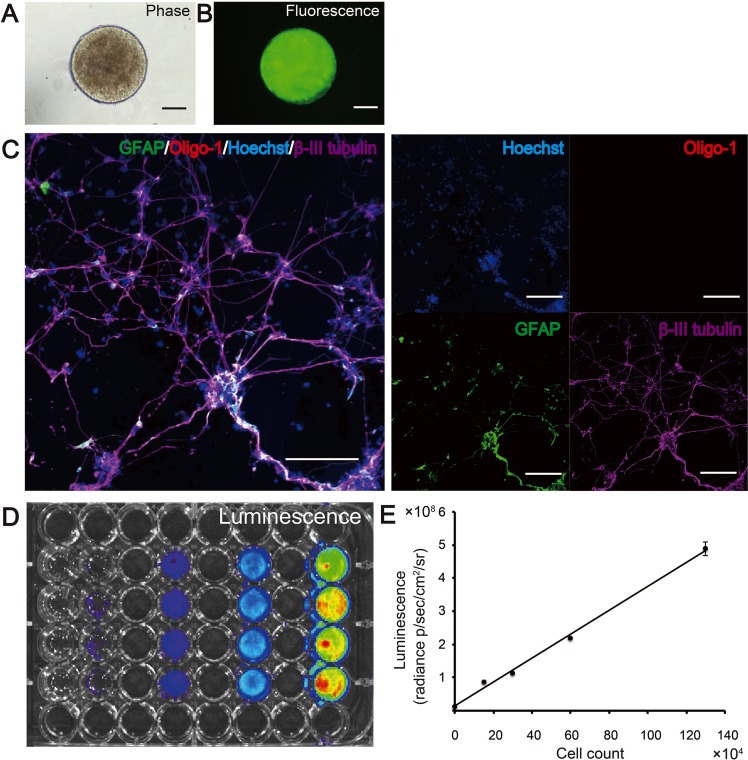
Detection of bioluminescence and fluorescence signals in lentivirally transfected 253G1-NS/PCs *in vitro*. Phase-contrast (A) and fluorescence (B) images of a neurosphere derived from tumorigenic 253G1 induced pluripotent stem cells. Neural stem/progenitor cells (NS/PCs) differentiated into β-III tubulin-positive neurons and glial fibrillary acidic protein (GFAP)-positive astrocytes *in vitro* (C). Bioluminescence imaging was used to detect bioluminescence signals in various numbers of 253G1-NS/PCs (0, 1.5 × 10^5^, 3 × 10^5^, 6 × 10^5^, and 1.2 × 10^6^ cells per well) (D). A direct linear correlation was found between cell numbers and photon counts *in vitro* (E). Scale bars in A–C, 1,000 μm.

hiPSC-derived NS/PCs generated from hiPSC clone 253G1 (253G1-NS/PCs) were cultured and labeled with the ffLuc gene (Venus fused to luciferase) [24] via lentiviral transduction (Fig. 1A, B). Differentiation assays revealed that the 253G1-NS/PCs differentiated into β-III tubulin-positive neurons and GFAP-positive astrocytes *in vitro*, but not into Oligo-1-positive oligodendrocyte progenitor cells (Fig. 1C). Sufficient fluorescence was detected from the 253G1-NS/PCs *in vitro* via fluorescence microscopy (Fig. 1D) for their subsequent identification *in vivo*. To examine the sensitivity of BLI, we used the Xenogen-IVIS system (Caliper Life-Sciences) to detect the luminescence intensity of the 253G1-NS/PCs at various cell numbers (ranging from 1.5 × 10^5^ to 1.2 × 10^6^ cells per well) in the presence of D-luciferin. Quantitative analysis of bioluminescent ffLuc signals revealed that the luminescence intensities were in direct proportion to the hiPSC-NS/PC numbers *in vitro* (r^2^ = 0.99) (Fig. 1D, E).

### Survival of hiPSC-NS/PCs grafted into the spinal cord of immunocompetent BALB/cA mice

**Fig 2:**
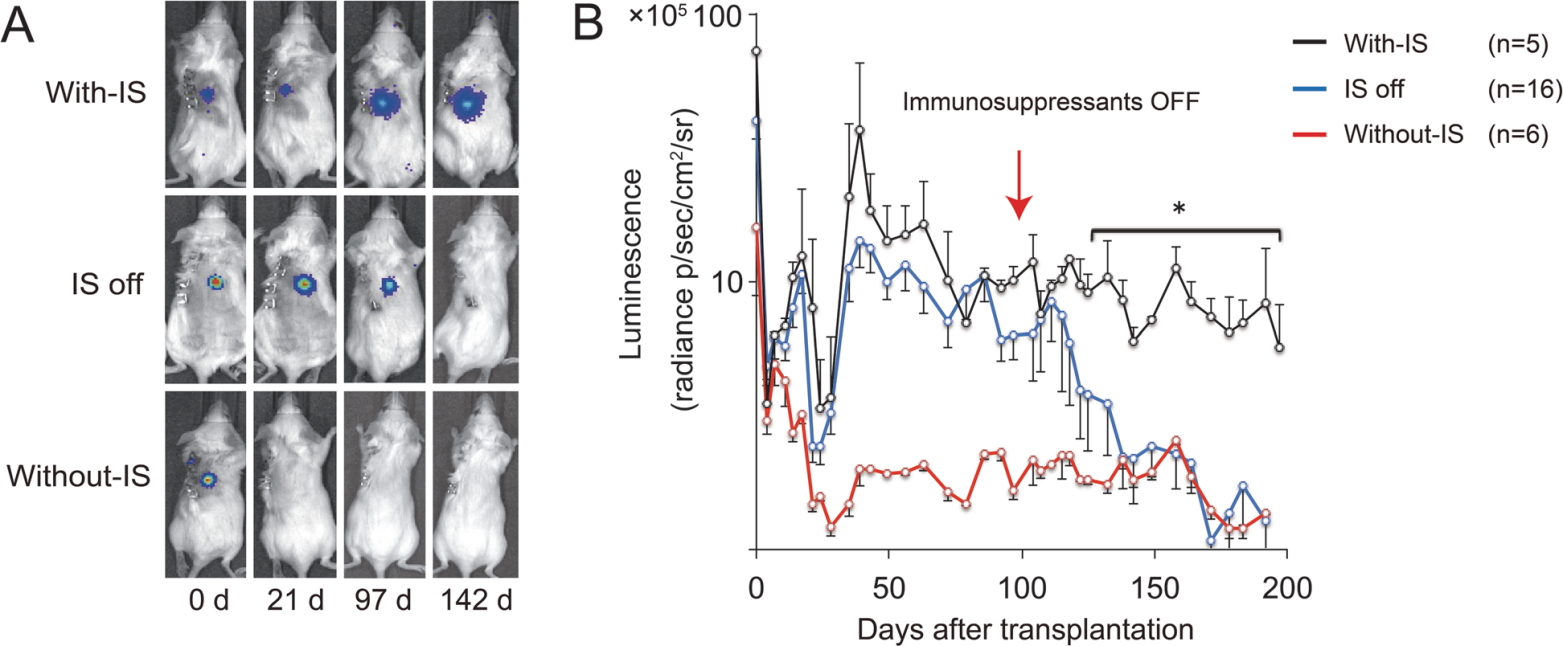
Representative *in vivo* images and quantitative analysis of photon counts derived from grafted hiPSC-NS/PCs. (A) Bioluminescence images of representative mice at 0, 21, 97, and 142 days after human induced pluripotent stem cell-derived neural stem/progenitor cell (hiPSC-NS/PC) transplantation. Upper panel: BALB/cA mouse with immunosuppressant treatment (FK506 plus anti-cluster of differentiation (CD) 4 monoclonal antibody (mAb); With-IS group); middle panel: BALB/cA mouse with immunosuppressant treatment, followed by discontinuation of immunosuppressants 100 days later (IS off group); lower panel: BALB/cA mouse without immunosuppressant treatment (Without-IS group). (B) Quantitative analysis of photon counts derived from grafted hiPSC-NS/PCs. Graft survival rate was 100% (n = 17/21, three animals died and one animal was sacrificed by day 21) in BALB/cA mice with immunosuppressant treatment (FK506 plus anti-CD4 mAb), versus 0% (n = 0/6) in BALB/cA mice without immunosuppressant treatment. After discontinuing the administration of FK506 and anti-CD4 mAb, all the grafted cells were rejected by day 164 and drastic reductions in signal intensity were observed. Data represent the mean value ± the standard error of the mean. (*p<0.05; Friedman’s test followed by Dunn’s post-hoc test.)

ffLuc-Transduced 253G1-NS/PCs were transplanted into the intact spinal cord of immunocompetent BALB/cA mice with immunosuppressants (FK506 plus anti-CD4 mAb; With-IS group, n = 21) and without immunosuppressants (Without-IS group, n = 6). The survival rate of the grafted 253G1-NS/PCs was then examined using *in vivo* BLI. The bioluminescent ffLuc signals emanating from the transduced 253G1-NS/PCs disappeared between 14 and 21 days after transplantation in the Without-IS mice (Table 1, Fig. 2A, B). This result suggests that immune rejection of the xenografted 253G1-NS/PCs occurred within 3 weeks in all of the mice without immunosuppressants, even though the spinal cord is a so-called immune-privileged site.

**Table 1:** Fate of hiPSC-NS/PCs transplanted into the mouse spinal cord.

		Graft survival						
Group	Immunosuppressant	0 d	28 d	72 d	123 d	138 d	148 d	200 d
**Without-IS**	**None**	6/6	0/3	0/2	0/2	0/2	0/2	0/2
		(100%)	(0%)	(0%)	(0%)	(0%)	(0%)	(0%)
**With-IS**	**FK506 + anti-CD4**	5/5	5/5	5/5	3/3	2/2	2/2	2/2
		(100%)	(100%)	(100%)	(100%)	(100%)	(100%)	(100%)
**IS off**	**FK506 + anti-CD4**	16/16	12/12	12/12	5/8	2/8	0/6	0/5
	**(Discontinued at 100 d)**	(100%)	(100%)	(100%)	(62.5%)	(25%)	(0%)	(0%)

Graft survival rate was 100% in the With-IS (FK506 plus anti-cluster of differentiation (CD4) monoclonal antibody) group, versus 0% in the Without-IS group. Immune rejection of transplanted human induced pluripotent stem cell-derived neural stem/progenitor cells (hiPSC-NS/PCs) was achieved by discontinuing the immunosuppressant treatment.

In the Without-IS group, three mice died by day 28 and one mouse was sacrificed by day 72. In the With-IS group, two mice died by day 138 and one mouse was sacrificed by day 123. In the IS off group, six mice died (four mice by day 28, a further one mouse by day 123, and a further one mouse by day 148) and five mice were sacrificed (three mice by day 123 and a further two mice by day 200).

**Fig 3:**
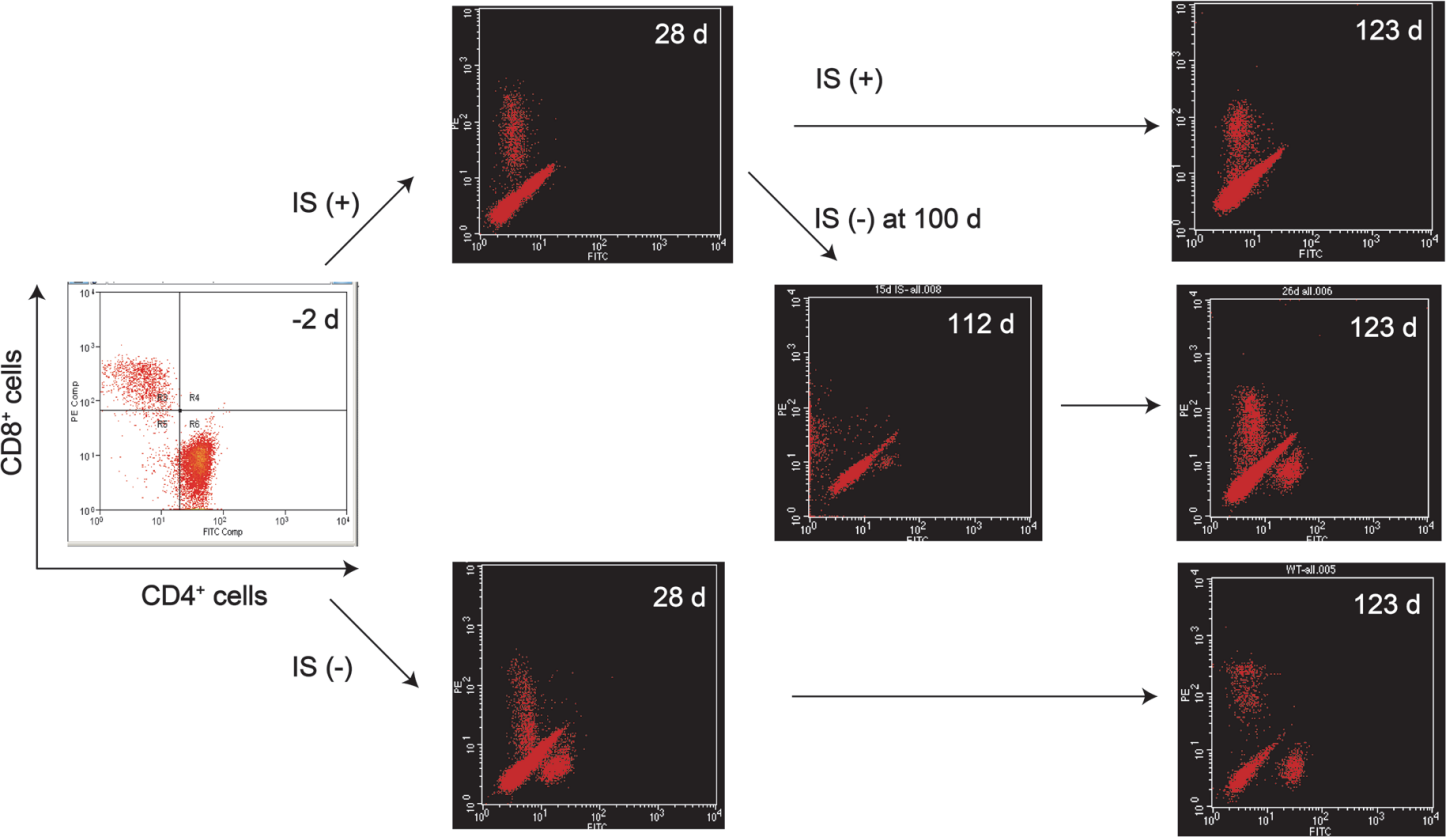
Lymphoid population in peripheral blood. Peripheral blood cells were analyzed using fluorescence-activated cell sorting-based flow cytometry. Data were gated on cluster of differentiation (CD) 4-positive or CD8-positive T-cell subsets. CD4-positive T-cells were depleted immediately after administration of FK506 plus anti-CD4 monoclonal antibody, but recovered after discontinuing immunosuppressant treatment. IS(+), with immunosuppressants; IS(−), without immunosuppressants.

The phylogenetic difference between mice and humans leads to a reduced affinity of murine T-cell receptors (TCRs) for human major histocompatibility complex (MHC) molecules. Therefore, immune recognition by an indirect pathway involving the host’s antigen-presenting cells and CD4-positive T-cells apparently participates in the elimination of the xenografted cells. Human iPSC-NS/PC rejection after cell transplantation is largely owing to a T-cell-mediated, donor-specific immune response [16,27–29]; therefore, we transplanted 253G1-NS/PCs in the presence of FK506 and anti-CD4 mAb immunosuppressants, as noted above, to avoid the immune rejection of the xenografts. Monitoring of the number of lymphocytes in the peripheral blood indicated that CD4-positive T-cells were depleted immediately after administration of the immunosuppressants but recovered at 28 days after drug discontinuation (Fig. 3).

Consistently, the long-term survival of the grafted 253G1-NS/PCs was achieved in all of the With-IS group mice through the use of immunosuppressants. The graft survival rate was 100% at 100 days after transplantation (n = 5/5 surviving animals), as well as at 200 days after transplantation (n = 2/2 surviving animals) (Table 1).

### Histological analysis of hiPSC-NS/PC-derived tumors

**Fig 4:**
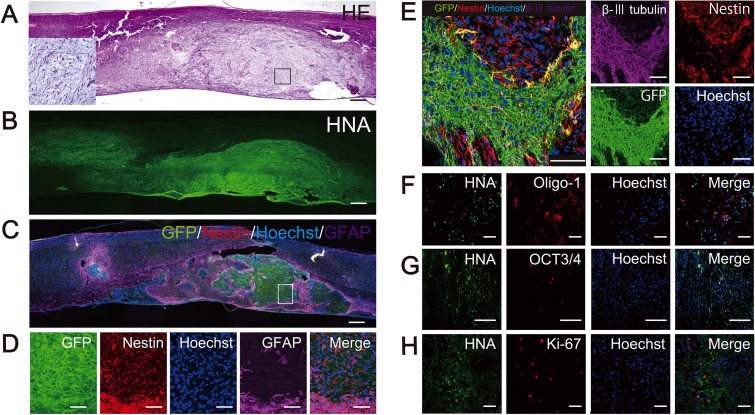
Tumor formation by grafted hiPSC-NS/PCs in the mouse spinal cord. Representative hematoxylin-eosin (HE)-stained (A) and human nuclear antigen (HNA)-stained (B) images of sagittal sections of spinal cord at 79 days after cell transplantation. HE staining revealed a biphasic tumor pattern with high and low cell density areas. The high cell density area contained compact bipolar cells with rosenthal fibers, whereas the low cell density area contained loose-textured multipolar cells with microcysts. The low cell density area surrounded by the square box is shown at higher magnification to the left of the image. Immunostaining for glial fibrillary acidic protein (GFAP), HNA, GFP, nestin, β-III tubulin (C, D, E), and Oligo-1 (F). The human induced pluripotent stem cell-derived neural stem/progenitor cell (hiPSC-NSC)-derived tumors mainly consisted of undifferentiated cells that stained positively for nestin, with low numbers of differentiated cells (e.g., β-III tubulin-positive neurons, GFAP-positive astrocytes, and Oligo-1-positive oligodendrocyte precursor cells). Nestin-positive cells were located in the center of the tumor, whereas differentiated cells were localized to the tumor margin (D, E, F). The boxed area in (C) corresponds to the higher magnification images in (D). Tumors contained a paucity of octamer-binding transcription factor (Oct) 4-positive (G) and Ki-67-positive (H) cells. The Ki-67 index was 7.0%. Scale bars in A–C, 500 μm; D–H, 100 μm.

The HE-stained spinal cord of a representative mouse in the With-IS group is shown in Fig. 4A. A number of HNA-positive (Fig. 4B) tumor cells occupied the entire spinal cord. The resultant tumors showed a biphasic pattern including high and low cell density areas. In the high cell density area, the tumor cells were characterized by small cell bodies with bland nuclei and elongated fibers. On the other hand, the tumor cells in the low cell density area were characterized by minute vacuoles and microcysts (Fig. 4A). Histologically, the tumors resembled low-grade glioma rather than teratomas (Fig. 4A, B).

The grafted 253G1-NS/PCs differentiated into β-III tubulin-positive neurons, GFAP-positive astrocytes, and Oligo-1-positive oligodendrocyte progenitor cells. In addition, we also observed cells that stained positively for Nestin, which is a neural progenitor marker, and octamer-binding transcription factor 4 (Oct4), which is a marker of undifferentiated pluripotent stem cells [30] (Fig. 4C–G). Cells also stained positively for the Ki-67 antigen, a marker of actively diving cells (Fig. 4H). The ratio of cells that stained positively for Ki-67 to cells that stained positively for Hoechst was 6.60 ± 0.22%.

### Recovery of CD4-positive T-cells, reconstitution of the host immune system, and immune rejection of hiPSC-NS/PC-derived tumors

To induce the ablation of the xenografted 253G1-NS/PC-derived tumors, we discontinued the immunosuppressant treatment (“IS off”) at 100 days after cell transplantation. Consistent with the recovery of CD4-positive T-cells (described above in Fig. 3), the rejection of the hiPSC-NS/PCs was initiated with the cessation of FK506 and anti-CD4 mAb administration. The graft survival rate gradually decreased to 62.5% (n = 5/8 surviving animals) at 123 days and 25% (n = 2/8 surviving animals) at 138 days (Table 1). At 164 days, the transplanted hiPSC-NS/PCs were completely ablated in all of the surviving mice (Fig. 2B). No tumor recurrence was detected for at least 200 days in the absence of immunosuppressants (Fig. 2B).

**Fig 5:**
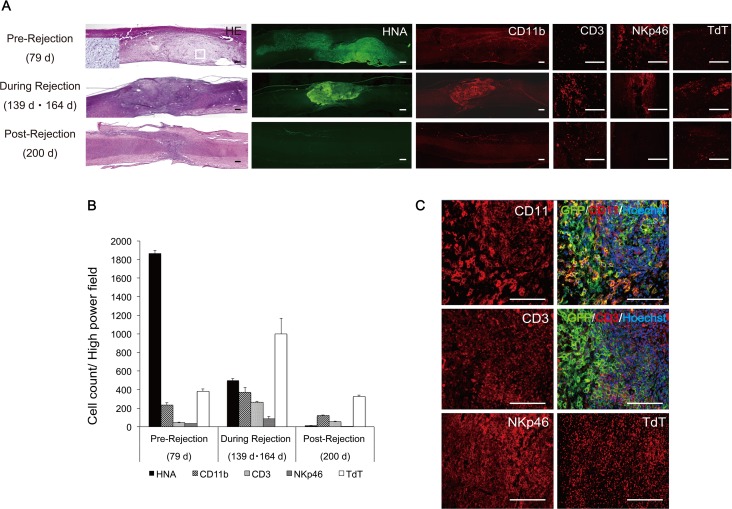
Analysis of inflammatory cell infiltration and apoptotic cells during the immune rejection of hiPSC-NS/PC-derived tumors. Immunohistochemical (A) and quantitative (B) analyses of human nuclear antigen (HNA)-, GFP-, cluster of differentiation (CD) 11b-, CD3-, NKp46-, and terminal deoxynucleotidyl transferase (TdT)-positive cells after discontinuing immunosuppressant treatment on day 100. After discontinuing the immunosuppressants, HNA-positive cells were gradually rejected. Inflammatory cells, including CD11b-, CD3-, and NKp46-positive cells, increased up to day 122 and then gradually decreased thereafter. TdT-positive cells also became prominent by day 122 and then gradually disappeared (C). Scale bars in A, 200 μm. hiPSC-NS/PC, human induced pluripotent stem cell-derived neural stem/progenitor cell.

Consistent with the BLI results, grafted hiPSC-NS/PC-derived tumors persisted in the immunocompetent BALB/cA mice with immunosuppressant treatment, but regressed after the cessation of FK506 and anti-CD4 mAb administration. During the course of immune rejection, CD11b-positive microglia, CD3-positive lymphocytes, and NKp46-positive natural killer (NK) T-cells infiltrated the tumors. TdT-positive cells were also observed within the tumors. Quantitative analyses of CD11b-positive, CD3-positive, NKp46-positive, and TdT-positive cells revealed that the numbers of all of these cells peaked at 122 days after iPSC-NS/PC transplantation (25 days after IS off), and then decreased at 164 days (64 days after IS off) to levels similar to those observed before immunosuppressant discontinuation (Fig. 5A–C).

## Discussion

### Rejection of hiPSC-NS/PC-derived tumors in a xenogeneic setting

Following the transplantation of 253G1-NS/PCs into the spinal cord of BALB/cA mice, essentially all of the grafted human cells were rejected within 3 weeks, despite the immune-privileged status of the spinal cord. The spinal cord in particular and the CNS in general are thought to be immunologically tolerant based on the following criteria: 1) the brain and the spinal cord are isolated from the rest of the body by the surrounding blood-brain barrier (BBB); 2) the CNS does not contain lymphatic vessels; 3) microglia and dendritic cells do not function normally in the CNS; and 4) low expression levels of MHC molecules are found in the CNS [12].

The expression levels of MHC class I molecules are also low on the surface of human ESC-derived NS/PCs and fetus-derived NS/PCs *in vitro*, whereas the expression levels of MHC class II molecules and co-stimulatory molecules are not detectable. Therefore, the immunogenicity of these NS/PCs is considered negligible. However, NS/PCs exert diverse immunomodulatory actions that increase the host CD4-/CD25-/Forkhead box P3-positive T-cell population, augment the levels of secreted immunomodulatory cytokines (e.g., interleukin (IL)-6), inhibit the presentation of antigens to host T-cells, and stimulate the activation and proliferation of host T-cells [18,31–36].

Notably, undifferentiated iPSCs can become immunogenic upon autografting [37]; however, the immunogenicity of iPSC-derived somatic cells can be different from that of undifferentiated iPSCs [4][38]. This is partly because the autografting study mentioned here utilized undifferentiated iPSCs and focused on immune responses against iPSC-generated teratomas rather than gliomas. Nevertheless, the *in vitro* differentiation of iPSCs into neural lineages prior to their transplantation is likely to prevent or at least attenuate their rejection by the host immune system [4].

Recent reports demonstrated that the immunogenicity of iPSCs and iPSC-derived products is as low as that of ESCs in syngeneic settings, whereas iPSCs become more immunogenic in allogeneic settings [38–42]. These reports were again directed toward immune responses against teratomas, as well as against skin grafts, and iPSC immunogenicity may differ in the context of the immune-privileged spinal cord. Furthermore, iPSC-NS/PC immunogenicity increases during the *in vivo* differentiation of NS/PCs in the CNS [43,44]. On the other hand, the NS/PCs utilized in the present study were differentiated *in vitro* and then xenografted into an immunosuppressed host, conditions under which the transplanted cells are less likely to be rejected [16]. Nonetheless, xenografted human NS/PCs were not rejected following their transplantation into a cerebral infarction animal model, even in the absence of immunosuppressants [45]. However, a critical weakness in the methodology of this previous study (i.e., the implanted cells were labeled with Hoechst 33342 prior to transplantation [45]) renders it difficult to draw clear conclusions about the survival of xenografts.

### Prevention of immune rejection of xenografted iPSC-NS/PC-derived tumors in immunosuppressed animals

According to previous reports [10,11], there is an optimal time window for the transplantation of cells in regenerative medicine strategies for SCI therapy. Considering the constraints of this time window (i.e., cell transplantation must be performed within several weeks after SCI), autologous transplantation upon the generation of host iPSCs and subsequent neural differentiation into NS/PCs is not a particularly realistic goal at this time. The situation, as it stands, necessitates the performance of allogeneic transplantation of iPSC stock-derived stem cells in clinical applications for SCI patients, along with combinatorial immunosuppressive treatment [4,46],[47].

Swijnenburg and colleagues used FK506 (tacrolimus), sirolimus, and mycophenolate mofetil (MMF) as immunosuppressant agents during the subcutaneous transplantation of human ESCs into mice [48]. FK506 monotherapy failed to support the successful engraftment of the xenografted cells, and combination therapy with MMF did not improve ESC survival. Similarly, the survival of xenografted hiPSC-NS/PCs was low when cyclosporin A was used as monotherapy [16]. Furthermore, our unpublished data revealed a low survival rate for xenografted hiPSC-NS/PCs in the mouse spinal cord when FK506, a clinically employed IL-2 blocker, was used as monotherapy (data not shown). For this reason, the current study used FK506 and anti-CD4 mAb as combination immunosuppressant therapy along with stem cell transplantation for SCI with reference to recent studies [49,50].

Antigen recognition is the first immunological barrier facing transplanted cells. Antigen recognition can be divided into two distinct pathways, direct and indirect recognition [51]. The indirect pathway is generally the most relevant in regenerative medicine applications. Hematopoietic stem cell transplantation, which occurs almost without the contamination of donor antigen-presenting cells, is the exception. The affinity of murine TCRs for human MHC molecules is low; therefore, it is reasonable to assume that transplanted human cells will be phagocytosed in murine antigen-presenting cells, followed by the presentation of human antigens to CD4-positive murine T-cells, and finally, the induction of the host immune response. In support of this idea, a study recently compared transgenic T-cell models of allogenic transplantation by grafting human ESCs into CD8-positive T-cell-knockout mice versus CD4-positive T-cell-knockout mice. The ESCs were immune rejected in both models, but the survival period was significantly longer in the CD4-positive T-cell-knockout mice than in the CD8-positive T-cell-knockout mice [52]. These observations provided the basis for our use of the anti-CD4 mAb together with FK506 in the present study. Indeed, the combination of these two immunosuppressants permitted the survival of unsafe hiPSC-NS/PCs in the mouse spinal cord for at least 200 days, with evidence of significant tumor growth in randomly selected spinal cord sections.

Auchincloss and Sachs suggested that the actions of NK cells are involved in immune rejection after organ transplantation [27]. However, transplantation of *in vitro*-differentiated ESCs is associated with increased expression of stem cell MHCs and decreased expression of ligands for NKG2D, an activating receptor found on NK cells [53–55]. Other investigations regarding allogeneic transplantation environments indicated that CD4-/CD8-positive T-cells play an important role in the immunity around the transplanted cells [20,52,56,57]. In agreement with these earlier reports, the present study showed that CD3-positive T-cells occupied a large proportion of the invasive hiPSC-NS/PC-derived tumors in the grafted mouse spinal cord, whereas there were fewer NKp46-positive cells.

The hiPSC-NS/PC-derived tumors observed in this investigation exhibited the histological features of low-grade, glioma-like tumor, with no endodermal or mesodermal components characteristic of teratomas. Histological analyses also revealed the differentiation of the transplanted cells into neurons, astrocytes, and oligodendrocyte precursors in the mouse spinal cord. However, the hiPSC-NS/PC-derived tumors consisted mainly of undifferentiated, Nestin-positive cells.

A previous report exploring the somatic cell origin of mouse iPSCs and their associated tumorigenicity showed that contamination of cell grafts with *Nanog*-GFP-positive cells increased the risk of teratoma formation [22]. However, the present study utilized hiPSCs derived from skin fibroblasts and failed to detect teratomas. Therefore, we considered that Oct4-positive cells rather than Nanog-positive cells might have been the cause of 253G1-NS/PC tumorigenicity. Oct4-positive cells were observed within the 253G1-NS/PC-derived tumors, suggesting that the transcription factor was reactivated within the invasive growths.

In addition, gene insertion by retroviruses, incomplete reprogramming of the somatic cells used to generate iPSCs, genomic instability owing to high iPSC passage numbers, and cell differentiation/induction methods must all be taken into account when considering the potential capability of iPSCs/iPSC-NS/PCs to form tumors. For example, mutations in the tumor suppressor gene in transplanted NS/PCs increased the risk of astrocytoma in a mouse model [58]. In addition, intracranial transplantation of neural precursors derived from transformed but not normal human ESCs yielded glioma in immunodeficient NOD/SCID mice [59]. Thus, further research is urgently required to shed light on the mechanisms of both teratoma and glioma generation following stem cell transplantation into the CNS.

### Tumor rejection following the discontinuation of immunosuppressants

Immunosuppressant treatment was discontinued in the present study at 100 days after iPSC-NS/PC transplantation. CD4-positive T-cells began to recover 12 days later, as assessed by FACS analysis, and ffLuc-positive hiPSC-NS/PCs gradually fell below the limit of BLI detection by 64 days after the cessation of immunosuppressants. The BLI technique cannot detect fewer than 1,000 cells; therefore, we also evaluated the presence of the transplanted stem cells by immunohistochemistry. At 64 days after FK506 and anti-CD4 mAb discontinuation, transplanted HNA-positive cells were still observed, but they were few in number and completely disappeared by 97 days. Two hundred days later, there was still no tumor recurrence.

Over the course of iPSC-NS/PC-derived tumor rejection, inflammatory cells (e.g., CD11b-positive cells, CD3-positive cells, and NKp46-positive cells) showed marked infiltration into the tumor. However, immune rejection with a focus on T-cells rather than NK cells was expected, based on the data described above. During the next phase of immune rejection, TdT-positive cells became prevalent, and then declined along with the numbers of transplanted cells and infiltrating immune cells.

As noted above, immune rejection following immunosuppressant discontinuation was hypothetically triggered by indirect antigen presentation (rather than MHC molecules) by hiPSC-NS/PCs and hiPSC-NS/PC-derived tumor cells to host T-cells. Nonetheless, regardless of the presence or absence of MHC molecules on the surface of the transplanted cells or their degree of differentiation, immune rejection occurred in all the transplanted cells. We postulate that other factors in addition to or instead of MHC molecules may be associated with the mechanism of immune rejection.

The BBB largely isolates the CNS from the rest of the body; therefore, inflammatory reactions rarely occur in the brain or the spinal cord without its collapse. However, even at 100 days after iPSC-NS/PC transplantation, immune rejection of the iPSC-NS/PC-derived tumor could be induced by the discontinuation of immunosuppressant treatment. It is possible that the tumor itself or our cell transplantation process stimulated breakdown of the BBB. For instance, earlier work showed that the use of a glass needle similar to the Hamilton syringe used herein for NS/PC transplantation disrupted the BBB. However, the BBB repaired itself in this earlier study within 12 days after cell transplantation, as evidenced by Evans blue dye transfer in rats[60]. Therefore, it is unlikely that transplantation of iPS-NS/PCs caused the continued collapse of the BBB at 100 days. Interestingly, another report indicated that activated lymphocytes can pass through the BBB[61], and that immune rejection can occur at 3 months after transplantation under xenograft settings.

The limitations of this study are as follows. The ablation of the tumor after iPS-NS/PC transplantation was observed in xenogeneic settings rather than allogeneic settings. The other limitation is that iPSC-NS/PCs were transplanted into the intact spinal cord rather than the injured spinal cord. In comparison, the immune response to allogeneic transplantation into the injured spinal cord greatly differs. Therefore, it must be determined whether the findings of the current study are replicated following allogeneic transplantation into the injured spinal cord. Furthermore, we must also examine the immunogenicity of iPSC-NS/PCs and the regulation of immune rejection. Additionally, the ultimate goal in cell replacement therapy is to minimize immune rejection and the use of immunosuppressants by matching HLA types between the donor cells and the host; however, it is practically difficult for iPSC banks to prepare iPSCs of all HLA types. Therefore, Yamanaka’s group is obtaining cells from a donor with homozygous alleles of the HLA-A, -B, and -DR loci to establish iPSC stocks in Japan. In terms of renal and bone marrow transplantation, survival rates would not be 100% even if grafts from a HLA 6 allele-matched donor were transplanted into an immunosuppressed recipient. Furthermore, it is unclear whether immune rejection of iPSC derivatives occurs in these settings. In addition, immune rejection might occur due to a mismatch between the donor and recipient in a locus other than HLA-A, -B, and -DR or in the minor histocompatibility antigen. Therefore, immunosuppressant treatment could still be necessary in current clinical trials using iPSC derivatives in Japan; however, because of their toxicity, we must consider minimizing the use of immunosuppressants. Recent clinical trials of fetal NSCs for SCI used transient immunosuppression. Assuming that transient immunosuppression is sufficient to graft iPSC derivatives, the discontinuation of immunosuppressant treatment will be time-limited. Moreover, tumorigenesis can occur even after the achievement of graft tolerance. In this case, other treatment strategies will be needed to ablate iPSC-derived tumors. Although other treatments, aside from surgery, have been proposed for brain and spinal cord tumors, including graft-derived tumors, none are curative. Immunotherapies have also been considered, but their effects are currently limited. In the present study, we induced the immune rejection of tumors derived from transplanted allogenic iPS-NSCs by discontinuing immunosuppressant treatment, which resulted in complete tumor rejection and no recurrence. This is the first report regarding the immune rejection of iPSC derivatives. Although there are various limitations, as mentioned above, this is a simple strategy with no side effects and should therefore be further tested as a means to ablate tumors following the transplantation of iPSC derivatives. We believe that these observations can lead to the control of the immune rejection of grafts and assist the realization of regenerative medicine.

## Conclusions

In this study, hiPSC-NS/PCs were xenografted into the spinal cords of immunosuppressed mice, resulting in the formation of tumors with the histological characteristics of low-grade gliomas rather than teratomas. The hiPSC-NS/PC-derived tumors were successfully rejected by discontinuation of immunosuppressant treatment. The issue of hiPSC-NS/PCs tumorigenicity remains a major concern in regenerative medicine applications; therefore, we propose that the discontinuation of immunosuppressants can provide a “safety lock” to ablate the grafted cells in the case of tumor development after stem cell transplantation therapy for SCI.

## Supporting Information

S1 ARRIVE Checklist (PDF)
